# Discovery of a Strongly-Interrelated Gene Network in Corals under Constant Darkness by Correlation Analysis after Wavelet Transform on Complex Network Model

**DOI:** 10.1371/journal.pone.0092434

**Published:** 2014-03-20

**Authors:** Longlong Liu, Jieqiong Qu, Xilong Zhou, Xuefeng Liu, Zhaobao Zhang, Xumin Wang, Tao Liu, Guiming Liu

**Affiliations:** 1 Ocean University of China, Qingdao, Shandong, People's Republic of China; 2 CAS Key Laboratory of Genome Sciences and Information, Beijing Institute of Genomics, Chinese Academy of Sciences, Beijing, People's Republic of China; 3 College of Pharmacy, Shandong University of Traditional Chinese Medicine, Jinan, People's Republic of China; Ecole Normale Supérieure de Lyon, France

## Abstract

Coral reefs occupy a relatively small portion of sea area, yet serve as a crucial source of biodiversity by establishing harmonious ecosystems with marine plants and animals. Previous researches mainly focused on screening several key genes induced by stress. Here we proposed a novel method—correlation analysis after wavelet transform of complex network model, to explore the effect of light on gene expression in the coral *Acropora millepora* based on microarray data. In this method, wavelet transform and the conception of complex network were adopted, and 50 key genes with large differences were finally captured, including both annotated genes and novel genes without accurate annotation. These results shed light on our understanding of coral's response toward light changes and the genome-wide interaction among genes under the control of biorhythm, and hence help us to better protect the coral reef ecosystems. Further studies are needed to explore how functional connections are related to structural connections, and how connectivity arises from the interactions within and between different systems. The method introduced in this study for analyzing microarray data will allow researchers to explore genome-wide interaction network with their own dataset and understand the relevant biological processes.

## Introduction

Coral reefs, by definition, are mainly three-dimensional shallow-water structures dominated by scleractinian corals [1]. Coral reefs occupy only a relatively small part of ocean acreages, yet harbor a considerably disproportionate amount of biodiversity, providing communities with ecosystem goods and services [2]. However, contemporary coral reefs constantly suffer from disease outbreak, overfishing, eutrophication, and ocean acidification. As a result, they increasingly fail to reassemble, leading to mass bleaching events [3], which marks the breakdown in the symbiosis with zooxanthellae [4]. The elevated coral mortality and resilience failure have caused persistent shifts from the original coral dominance to algal preponderance [1], [5], [6].

Numerous efforts have been made, not only by local residents but also by scientists, to achieve the overall goal of management of coral reefs, on which the human welfare depends [7]. Accompanied by increasingly faster sequencing technology and lower cost, molecular level studies will be beneficial to coral reef conservation and management, such as barcoding [8], microarrays [9]–[13] and comparative genomics [14], [15]. Levy et al. [13] performed microarray analysis of coral genes with *A*. *millepora*, and indicated that many genes involved in stress responses and protection were coupled to the circadian clock. In their study, signals from time domain were transformed into frequency domain signals using the FFT (fast Fourier transform) method.

This study analyzed a published data set from Levy et al. [13] with a novel method—correlation analysis after wavelet transform on complex network model, to explore the effect of light on gene expression in *A. millepora*. Wavelet transform, of which the time-frequency resolution depends on the signal frequency, can be used for multi-scale analysis of signal through dilation and translation, so it can effectively extract time–frequency features [16]. At high frequencies, the wavelet reaches at high time resolution but low frequency resolution; whereas, at low frequencies, low time resolution and high frequency resolution can be obtained [17]. By the advantage of multi-resolution, we can locate time and frequency more accurately. Firstly, dimension reduction was achieved by Euclidean distance clustering. Secondly, wavelet transform of 300 probes sampled through time axle was conducted. Finally, 50 key genes with large differences were screened, annotated and analyzed. In this way, we identified important genes including both previously-published genes with experiment support and novel genes without accurate annotation, which provides useful information for future research. Moreover, network characterization of gene connectivity under constant darkness shed light on our understanding of the coral's response toward light changes and an orchestrated correlation among genes under the control of biorhythm. Our method has shown advantages in dealing with extremely-complicated time serial data, which would be of wide use for similar data analyses.

## Materials and Methods

### Data generation

The data set used in this study was published by Levy et al. [13], available online in the NCBI database (GEO: GSE21658, Platform ID: GPL6941). The original aim of this data set was to better understand how corals tune their circadian machinery to respond to external and internal cues. According to the experimental conditions, 2 groups were divided as LD group (alternate light and dark) and DD group (continuous dark). Three coral colonies were sampled at 4 hr intervals during 2 consecutive days under an ambient LD cycle or under DD. A total of 72 arrays (GSM540430-GSM540501) were hybridized (n = 3) and the number of probes was 18432 in each array.

In our study, the average of 3 biological replicates' was used, obtaining a total of 24 groups of data. For each probe, there was a 12-dimensional vector composed of its gene expression data taken at 12 time points under the same experimental condition, populating original databases (Table S1). The number of records in original databases was reduced from 18432 to 17198 after deleting those with no corresponding genes (324) and averaging those multiple probes for a single gene. To avoid the immoderate impact of the differences on the model, each record was normalized to [−1, 1] using the following formulae:

If *x*_min_ ≠ *x*_max_, then
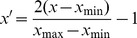
if *x*_min_  =  *x*_max_, then
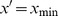
where *x* and *x′* represent vector before and after normalization; *x*_min_ and *x*_max_ represent the minimum and maximum of the vectors' elements before normalization, respectively. Working databases were established via data preprocessing (Table S2).

### Capture of important genes by Euclidean distance clustering analysis

**Figure 1:**
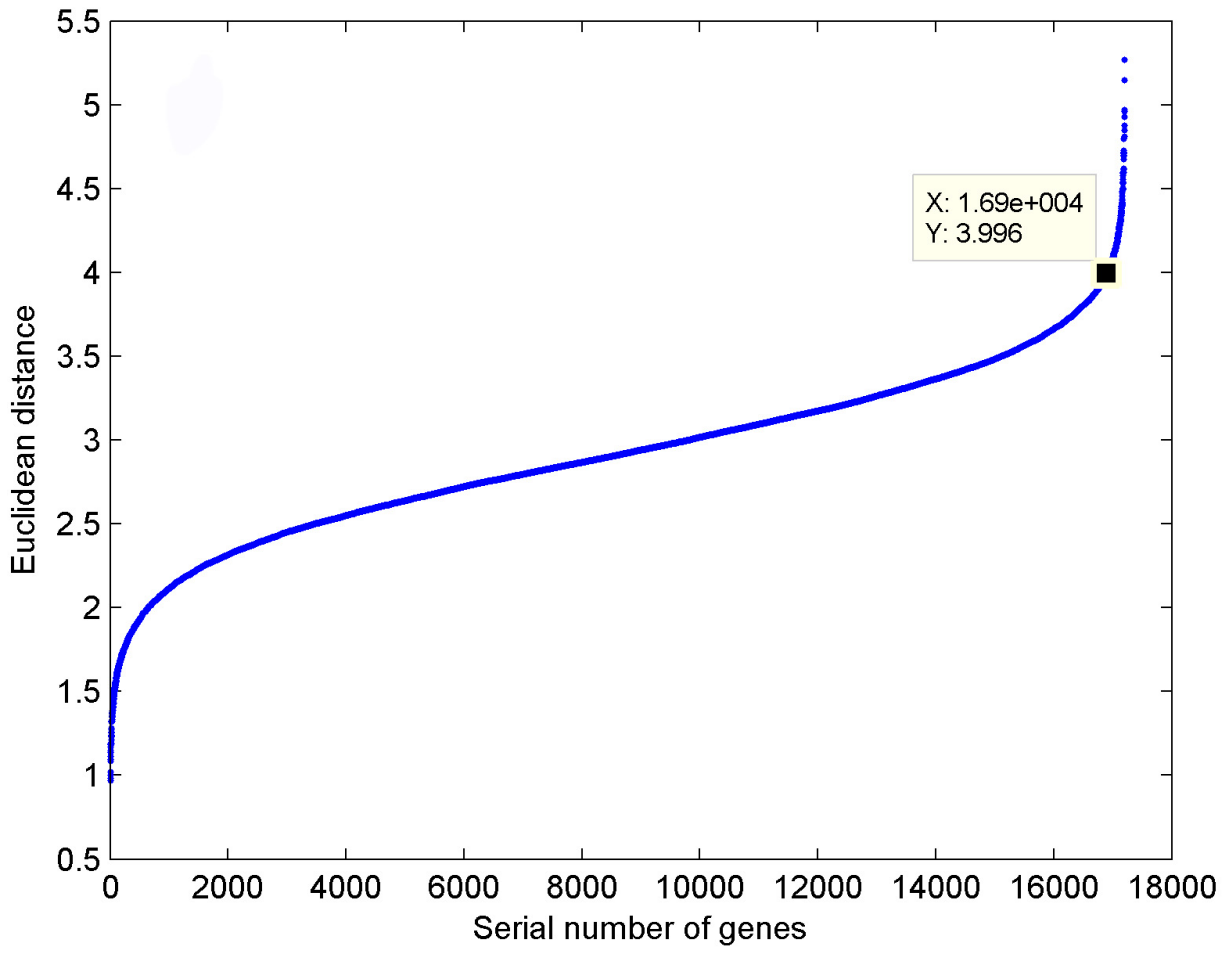
Scatter diagrams of the Euclidean distances of the probes' expression data between the 2 groups.

To explore the effect of light on gene expression, genes with large expression differences between the two groups were considered key genes significantly affected by light. Expression differences were measured by Euclidean distance, using the following formula:
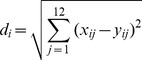
where *x_ij_* and *y_ij_* represented the values of element *j* of record *i* in DD and LD groups respectively. Figure 1 shows the Euclidean distances of the 17198 probes between two groups. After smoothing, an inflection point (16898, 3.996) was found using MATLAB. For further analyses, the 300 probes were selected as important genes (Table S3).

### Wavelet transform

Expression data at discrete time points are non-stationary signals containing a variety of frequency components. A discrete wavelet transform was adopted to process the expression data, and was performed according to the following formulae:
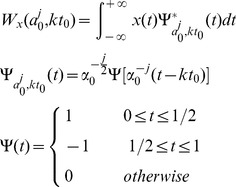
where *α*_0_ = 2, *t*_0_ = 2, *j* = 0,1;2,…, *k* = *N*, *N* denoted the set of natural numbers, 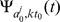 represented the wavelet basis function, and 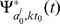 was the conjugate function of 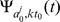. Here, 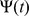 was defined as the wavelet function. The Mallat algorithm in the MATLAB toolbox was used to achieve wavelet transform for each probe record *X_i_* = (*x_i1_*,…,*x_ij_*,…,*x_i12_*), where *i* = 1,…,300; *j* = 1,…, 12. The approximate signals *C_i_* = (*c_i1_*,…,*c_ij_*,…,*c_i6_*) and the detailed signals *D_i_* = (*d_i1_*,…,*d_ij_*,…,*d_i6_*) of each probe record were acquired according to the following formulae:
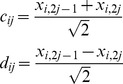
where *i* = 1,…,300, *j* = 1,…,6.

The frequency vector of each probe was composed of an approximate signal, while the detailed signals constituted a 12–dimensional vector. The frequency vectors of probe *i* were 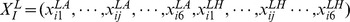 and 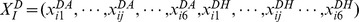 in LD and DD groups respectively, where the superscripts *LA* and *LH* represented the low- and high-frequency coefficients of the probe expression data in the LD group, and the superscripts *DA* and *DH* represented the low- and high-frequency coefficients of the expression data in the DD group (Table S4).

### Correlation analysis in the frequency domain

A correlation analysis quantitatively analyzed the similarity and interdependence of 300 probes in the frequency domain between the two groups. The specific algorithm was as follows:

(1) Covariance of the frequency vectors *X_i_* of probe *i* and *X_j_* of probe *j*.
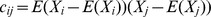



(2) Correlation coefficient between the frequency vectors *X_i_* and *X_j_*.
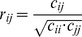



The correlation matrix *R* = (*r_ij_*)_300×300_ was thus obtained, which was a real symmetric matrix. We then defined the connectivity of a probe to measure the number of highly-relevant probes (0.7≤|*r_ij_*|≤1). The connectivity of probe *i* was defined as the number of probes that were highly relevant to probe *i*.

### The establishment of complex gene network

The connectivity of a probe (gene) to certain extent reflected its importance in the whole gene network, i.e., the larger the connectivity, the higher the importance. Through comparing the differences in connectivity between the 2 networks (LD and DD groups), we then identified those probes with remarkable changes in connectivity.

The algorithm to calculate the connectivity was as follows:

(1) Defining a connectivity matrix *L* = (*l_ij_*)_300×300_, where *l_ij_* represented the connectivity between probe *i* and probe *j*.

(2) In the matrix *R* = (*r_ij_*)_300×300_, if 0.7≤|*r_ij_*|≤1, then *l_ij_* = 1; else, *l_ij_* = 0.

(3) Ignoring autocorrelation, i.e., *l_ii_* = 0.

(4) Calculating the connectivity *l_i_* of probe *i*,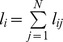, *i* = 1,…,300, where *N* = 300.

(5) The difference of the connectivity of probe *i* between LD and DD groups was obtained by subtracting the connectivity 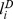 in the DD group from the connectivity 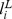 in the LD group:
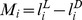



The detailed information about *M* is listed in Table S5, and an example of calculating the connectivity (probe B015-C2) is illustrated in Figure S1.

In this way, we discovered 168 probes whose connectivity was greater in the DD group than in the LD group, whereas there were 119 probes whose connectivity was greater in the LD group than in the DD group. The square ratio of negative or positive difference was defined as follows:
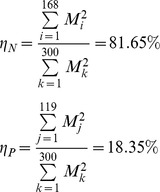



Here, *i* and *j* represent probes with negative and positive differences respectively.

**Figure 2:**
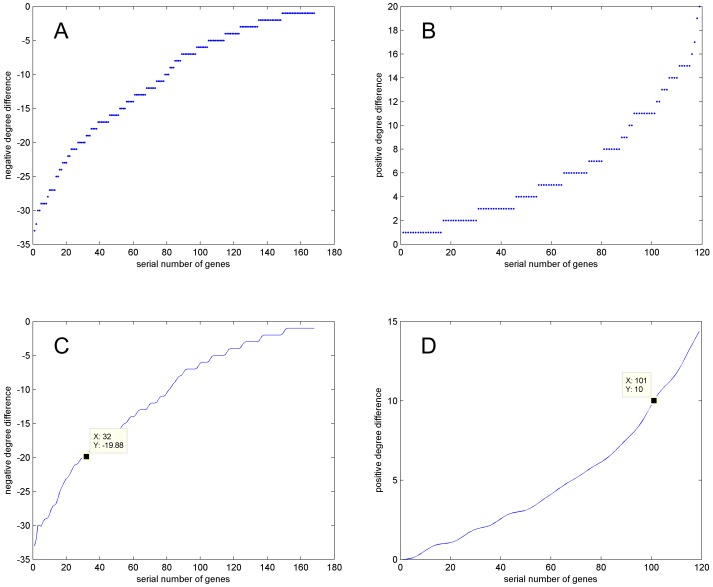
Difference of connectivity between LD group and DD group. A: Negative difference obtained by subtracting degree numbers of probes in DD group from degree numbers of corresponding probes in LD group. B: Positive difference gained throughout subtracting degree numbers of probes in DD group from degree numbers of corresponding probes in LD group. C: Negative difference curve by smoothing scatter diagram of negative difference in A. D: Positive difference curve by smoothing scatter diagram of positive difference in B.

The curve in Figure 2A was smoothed, and its inflection point (32, −19.88) in Fig. 2B was obtained using MATLAB. In the same way, another inflection point (101, 10) was also identified (Figure 2B). As shown in Table S6, a total of 18 probes with large positive differences and 32 probes with large negative differences were selected from inflection points.

### Probe annotation and analysis

All the 50 probes were believed to be key genes and annotated in GO database (http://amigo.geneontology.org/cgi-bin/amigo/blast.cgi?). After filtering the *P* value of 1.0e-5, 11 genes (61.11%) with large positive differences (Table 1) and 19 genes (59.38%) with large negative differences (Table 2) were finally obtained.

**Table 1:** Annotated differentially-expressed probes with positive difference.

GenBank acc.	Annotation by GO BLAST Search	*P*-value
DY582337	NADH dehydrogenase (ubiquinone) Fe-S protein 6 [18]	2.0e-26
DY584986	protein tyrosine phosphatase, receptor type, s, a	5.6e-13
DY584757	ATP synthase subunit alpha	3.1e-91
DY577937	actin-related protein	4.0e-69
GO004138	Peptidyl-prolyl cis-trans isomerase	7.0e-47
DY582070	ubiquitin B	3.8e-137
GO002856	seryl-tRNA synthetase	3.6e-91
GO003374	Transcriptional regulator ERG	3.7e-26
DY579678	phosphatidylinositol-specific phospholipase C [19]	3.3e-33
DY582316	Mediator of RNA polymerase II transcription subunit 30	1.9e-09
DY579711	Rae1/Gle2	5.8e-24

**Table 2:** Annotated differentially-expressed probes with negative difference.

GenBank acc.	Annotation by GO BLAST Search	*P*-value
DY582598	G2/mitotic-specific cyclin-B2 [20]	2.0e-60
DY580293	RNA binding motif protein 4B	6.9e-16
GO003570	U2 snRNP-associated SURP motif-containing protein	2.5e-15
DY582400	cyclin B2 [21]	3.0e-41
DY580978	tyrosine 3-monooxygenase/tryptophan 5-monooxygenase activation protein [22], [23]	3.2e-63
DY580776	Epsin-2	1.1e-73
DY580431	phosphatidylinositol transfer protein, beta	1.5e-63
GO000385	Thymosin beta-10	7.5e-11
DY578730	WD repeat-containing protein 1/actin interacting protein 1	1.4e-07
DY582473	WW domain-binding protein 4	3.7e-34
DY580643	monooxygenase, DBH-like 1	9.4e-11
DY578693	Animal heme peroxidases and related proteins	2.7e-18
DY577894	Transcriptional repressor protein YY1	1.2e-55
DY578322	tetraspanin 11	1.5e-22
DY586112	60S acidic ribosomal protein P1	6.1e-18
DY577919	Membrane magnesium transporter 1	3.4e-15
DY584166	heme binding protein 2 [24], [25]	4.7e-36
DY583753	proteasome subunit, alpha type, 4	1.7e-105
DY582625	Pre-mRNA-splicing factor 38B	5.0e-74

## Results

In this study, 18 probes with large positive differences and 32 probes with large negative differences were selected (Table S6). After gene annotation, we finally obtained 11 genes (61.11%) with large positive differences and 19 genes (59.38%) with large negative differences. The square ratio of negative differences (81.65%) was much larger than the square ratio of positive differences (18.35%), which can be explained by that more genes were upregulated coincidently in corals under LD cycles to maintain normal life activities; when under DD, the previous gene network was weakened, and the corresponding genes were suppressed for the life activities of the coral-algal symbiotic system.

Within the positive group (Table 1), probes with fundamental functions were listed, with some believed to be house-keeping genes. Ubiquinone (DY582337) functions as a component of the mitochondrial respiratory chain, while protein tyrosine phosphatase (DY584986) functions in a coordinated manner with protein tyrosine kinases to control signaling pathways. ATP synthase (DY584757) synthesizes cellular ATP from ADP and inorganic phosphate, while seryl-tRNA synthetase (GO002856) belongs to the category of tRNA synthetase. Besides, actin (DY577937) and ubiquitin (DY582070) are usually regarded as reference genes, whose expressions remain stable under different conditions. Our results indicated that these key genes worked synchronically and formed a closer network to maintain homeostasis under an ambient LD cycle, whereas this network became looser and disordered under DD, which is partly in agreement with Levy et al. [13] who suggested that coordinated chaperone expression probably reflected the diel pattern of stress experienced by symbiotic corals. In short, these genes formed a synchronous alliance in basal metabolism, keeping the whole organism functioning well under normal conditions.

For the negative group listed in Table 2, some genes are proven to be related to light response or cell cycle. DY584166 was previously annotated as heme binding protein 2, which was certified to be light-regulated gene in zebrafish [25]. DY582598, DY582400 and DY577894 were annotated as cyclin B2 or related proteins, which regulate the G2-M transition [21]. With the absence of light in DD group, circadian clocks might be disturbed, resulting in adjustment of cell cycle, which was the case for plants [26]. Although such assumption lacks support from existing document and experiment, we believe that it is reasonable, because rhythm control may work as a whole, with related genes interacting with each other.

## Discussion

Some researchers believed that environmental stimuli could not result in so complex nonlinear dynamical systems such as heart rate and blood pressure [27], [28]. However, a growing body of evidences has shed light on the complicated nature of organism's reaction towards ecological stimuli—complex gene behavior, including upregulated or downregulated expression, and sometimes gene silencing. Due to the combinatorial complexity of gene interaction networks, it is difficult to identify key genes or coordinated gene pairs, and numerous investigative methods have been developed and used, e.g. weighted network analysis, hierarchical model-based analysis and nonlinear multivariate prediction [29].

However, few methods were applied in reef-building coral related researches. In the era of big data, data mining of complex gene network is crucial to understand complicated behavior of an organism. The thought of complex network has been widely used in brain connectivity studies and has been proven to be reliable [30], easily computable [31], [32], and useful for exploring structural–functional connectivity relationships [33]. Here we intended to explore the effect of light on gene expression in *A. millepora* by correlation analysis after wavelet transform on complex network model. Euclidean distance clustering analysis and the ‘hard’ threshold strategy were used after normalization of the original data. During the whole process of data analysis, wavelet transform played a decisive role. Previously, wavelet transform was mainly applied in image coding, multisensor image fusion and texture classification. As there were no precedents in applying wavelet transform in microarray analysis, the present study for the first time used and proved this method to be effective and accurate in analysis of microarray data. Wavelet transform has advantage over other methods, for it providing an adjustable time-frequency window that can automatically narrow to improve the time resolution in the high-frequency region and broaden to improve the frequency resolution in the low-frequency region [17]; therefore, detailed signals with several closely-related genes and approximate signals with hundreds of genes were obtained. In this way, certain inconspicuous characters of genes can be made distinctive and captured. We tried direct data processing without wavelet transform and only obtained less than 10 key genes with significant differences (data not shown). Comparing with other studies, the present work showed a high efficiency, with 60% (30/50) obtained differentially-expressed genes successfully annotated. For example, only 22% of the 309 differentially-expressed genes identified using maanova method have annotations [12].

A microarray is a complex biochemical-optical system whose purpose is to simultaneously measure the expression of thousands of genes, and thus can be regarded as a complex gene network. The topology of a microarray can be determined using different statistical or biological criteria. A few nodes can play a vital role in maintaining the network's connectivity [34]. When it comes to the gene network, these nodes (genes) may share the same co-expression pattern or cooperate tightly in cellular processes that lay a foundation for subsequent activities. It is thought that the growth of a network in which new nodes are preferentially attached to already established nodes may also be used to characterize the evolution of biological systems [35], [36]. Under DD, reef-building corals in this study tended to build a tighter gene network, as evidenced by probes with large negative differences. In other words, these genes were tightly correlated to fight against abnormal condition (i.e., DD).

Key genes are important but they often demonstrate distinctively different patterns of expression, which is hard to apply to overall management. Viewing the gene activities from the aspect of complex network, the present study provided a genome-scale overview of coral's response toward light changes and revealed an orchestrated collaboration among genes under the control of biorhythm. Network characterization of gene connectivity is useful not only for exploring gene co-expression, but also for revealing abnormality in physiological disorders. Therefore, the present data provide us a theoretical basis to better protect the coral reef ecosystems.

This network-scale analysis of coral microarray data demonstrates a possibility to investigate gene interaction in corals under different light conditions. A group of genes are screened, showing closer relationship in fighting against continuous darkness. These genes are the key nodes in gene network for coral activity towards light. Our next study will explore how functional connections are related to structural connections, how connectivity arises from interactions within and between different systems and the underlying principles. We believe that the method introduced in this study will be of wide use for researchers to further explore their own datasets and better understand the underlying biological mechanisms.

## Supporting Information

Figure S1 **An example of calculating the connectivity.** There were only 7 probes which were highly relevant to the probe B015-C2 in the LD group (a), while there were 37 probes highly relevant to B015-C2 in the DD group (b). This implied that B015-C2 was more important in the DD group than in the LD group. Later probe annotation indicated that B015-C2 was related to cell mitosis.
(JPG)

Table S1 **Two original databases of all probes in the DD and LD groups.**
(XLS)

Table S2 **Working databases established via data preprocessing original databases.**
(XLS)

Table S3 **Expression data of 300 important probes with large differences of expression.**
(XLS)

Table S4 **Frequency coefficients of 300 important probes expression data in the DD and LD groups.**
(XLS)

Table S5 **The results of correlation analysis in frequency domain for 300 important probes.**
(XLS)

Table S6 **Detailed information of 18 probes with large positive degree differences and 32 probes with large negative degree differences.**
(XLS)
