# Supplementary figures and images for: Discovery of a Strongly-Interrelated Gene Network in Corals under Constant Darkness by Correlation Analysis after Wavelet Transform on Complex Network Model
Source: PLoS One. 2014 Mar 20;9(3):e92434. doi: 10.1371/journal.pone.0092434 (PMC3961355; doi:10.1371/journal.pone.0092434)

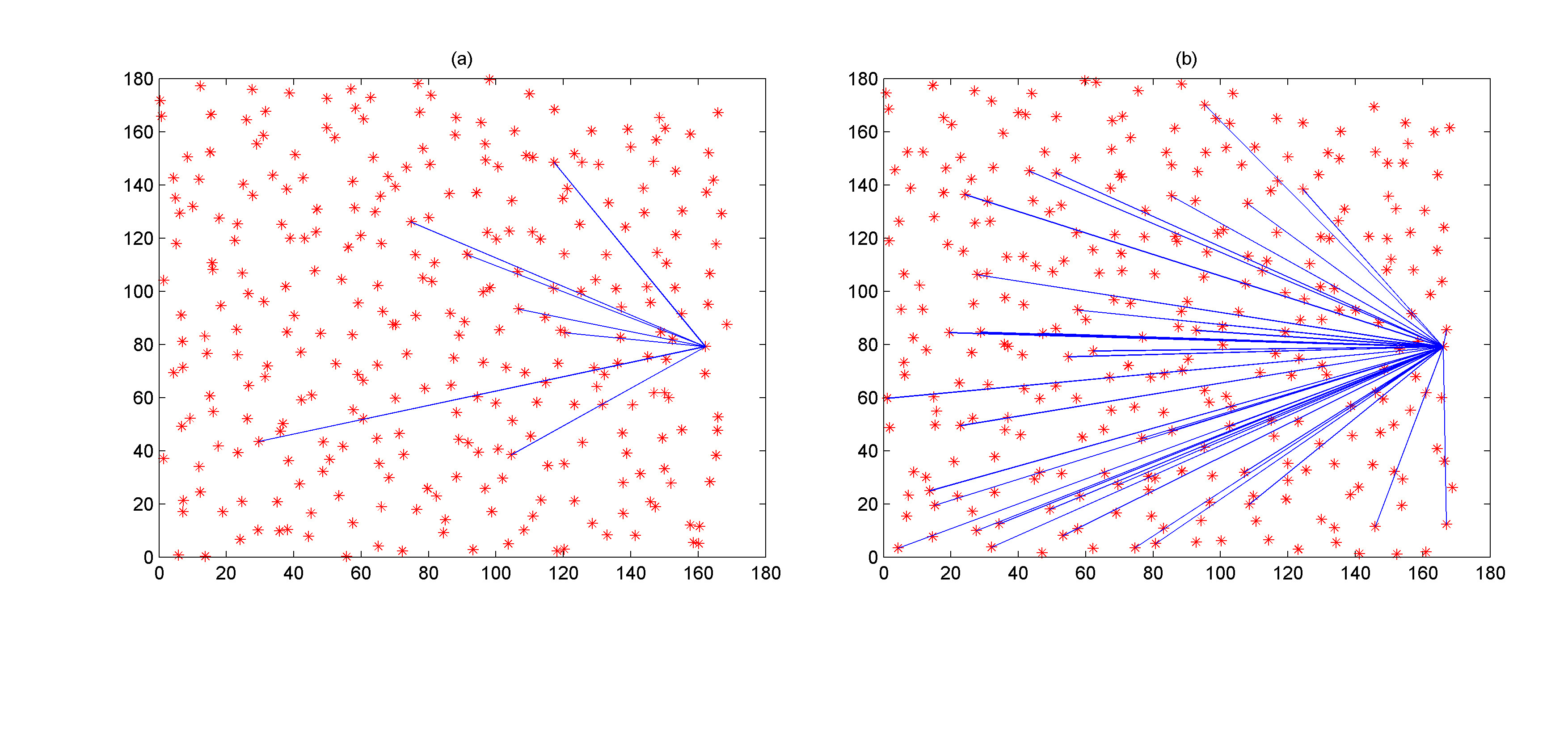

Supplement: Figure S1 — An example of calculating the connectivity. There were only 7 probes which were highly relevant to the probe B015-C2 in the LD group (a), while there were 37 probes highly relevant to B015-C2 in the DD group (b). This implied that B015-C2 was more important in the DD group than in the LD group. Later probe annotation indicated that B015-C2 was related to cell mitosis. (JPG) [file pone.0092434.s001.jpg]
